# Frozen Natural Orbitals‐Based Coupled‐Cluster Singles, Doubles, and (full) Triples ‐ A Computational Study

**DOI:** 10.1002/asia.202500472

**Published:** 2025-06-06

**Authors:** Manisha, Prashant Uday Manohar

**Affiliations:** ^1^ Department of Chemistry BITS‐PILANI Pilani campus Pilani 333031 India

**Keywords:** CCSDT, FNO, Force constant, Single precision, Triplet–singlet gaps, Vibrational frequency, XFNO

## Abstract

Frozen (F) natural orbitals (NO) approach in coupled cluster (CC) singles and doubles (SD) and equation‐of‐motion (EOM) CCSD methods is well‐known for provide cost‐effective yet accurate alternative for energy computation. In this article, we extend the FNO approach to CCSDT (CC with singles, doubles, and triples) implemented within *Q‐CHEM*. This can be employed within both the (conventional) double precision (DP) as well as the single precision (SP) algorithms. Errors due to employing SP algorithm instead of DP are insignificant and therefore are not discussed. However, for computational timings, we present the performance of FNO‐CCSDT versus conventional CCSDT methods with both SP and DP algorithms using water molecule as a test system. FNO‐CCSDT results at different thresholds can be extrapolated to give the XFNO‐CCSDT approach, which provides an enhanced accuracy. To illustrate this, we present total energies of a few molecules, adiabatic triplet–singlet gaps of a few chromophores and bond‐stretching trends in total energies and vertical triplet–singlet gaps of hydrogen fluoride molecule. We also examine these methods for numerical estimation of spectroscopic parameters – force constants and vibrational frequencies of some diatomic molecules.

## Graphical abstract

The frozen natural orbitals (FNO)‐based CCSDT is a cost‐effective approach and provides significant computational speed‐up with rather insignificant errors (standard deviation ∼0.9 millihartrees) – smaller than the CCSDT accuracy limit of ∼1.5 millihartrees. Extrapolation of FNO‐CCSDT energies computed using different occupation thresholds results in the XFNO‐CCSDT method, which has more balanced accuracy with a standard deviation of ∼0.6 millihartrees.

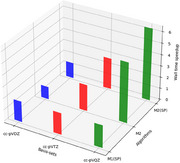

## 1 Introduction

Electron correlation^[^ ^1^ ^]^ is indispensable for accurate electronic structure computation of molecules. Amongst the available electronic structure methods, the CC,^[^ ^2^, ^3^, ^4^, ^5^ ^]^ and in particular, the CCSD^[^ ^6^, ^7^, ^8^ ^]^ have evolved over the last few decades as black‐box tools in most of the quantum chemistry packages^[^ ^9^, ^10^, ^11^ ^]^ for studying bonding‐patterns, energies, energy‐gaps, properties, and thermochemistry of molecules. The exponential nature of the wave‐operator in CC ansatz, along with the connectedness of CC equations, not only guarantees the size‐extensivity (and size‐consistency – provided that the reference wavefunction too, is size‐consistent), but also incorporates important contributions from all orders of perturbation resulting in high accuracy. The success of CCSD in efficiently computing the ground state energy and properties of molecules boosted the research in extending its applicability to excited states and ionized/electron‐attached states, which exhibit strong near‐degeneracy. This led to emergence of methods, which can be broadly classified into two categories, namely, the single reference (SR)^[^ ^12^, ^13^, ^14^, ^15^, ^16^ ^]^ CC and the multi‐reference (MR)^[^ ^17^, ^18^, ^19^, ^20^, ^21^, ^22^, ^23^ ^]^ approaches, and differ in the way they incorporate the static and dynamic electron correlation effects. For example, Fock‐space (FS)CC,^[^ ^17^, ^18^, ^20^ ^]^ Hilbert space (HS)CC,^[^ ^21^, ^22^ ^]^ intermediate Hamiltonian‐based (IH)FSCC,^[^ ^23^ ^]^ etc. use a set of configurations to describe the reference wavefunction in order to effectively incorporate the static correlation. The dynamic correlation is then brought in by action of a sui wave‐operator on this multi‐ configurational wave‐function and thus, these methods are referred to as MRCC methods. On the other hand, equation‐of‐motion (EOM)CC,^[^ ^15^, ^16^, ^24^, ^25^ ^]^ linear‐response (LR)CC,^[^ ^26^ ^]^ etc. can effectively handle the degeneracies in the electronic states in a simple SR framework, making them more popular over the MR methods.[Supplementary-material asia202500472-supl-0001]

The aforementioned MR and SR CCSD approaches yield an accuracy of 5∼10 kcal ${\mathrm{mol}}^{-1}$ ^[^ ^27^, ^28^ ^]^ in the excitation energies with a scaling of $O(N^{6})$, where N is the computational size of the system. Clearly, further accuracy, although desirable, is a bit challenging to achieve. The intermediate cost‐effective attempts in improving the CCSD wavefunction span variety of methods that bring in the (partial) effects of triples perturbatively, such as CCSD(f/dT),^[^ ^29^ ^]^ CCSD(2),^[^ ^30^ ^]^ or the more popular CCSD(T) (often referred to as gold standard in quantum chemistry)^[^ ^31^, ^32^ ^]^ and can yield the accuracy of 1∼2 kcal ${\mathrm{mol}}^{-1}$ for total energies with a scaling of $O(N^{7})$. However, due to non‐iterative nature of these methods, the correlation effects may not be balanced across multiple electronic states, resulting in larger errors in energy‐gaps^[^ ^33^ ^]^ relative to CCSD. Balanced description of correlation effects can only be achieved by including full triples, that is, the CCSDT^[^ ^34^ ^]^ method which provides the accuracy of 0.5∼1 kcal ${\mathrm{mol}}^{-1}$ for total energies and of 0.3∼0.5 kcal ${\mathrm{mol}}^{-1}$ for energy differences^[^ ^35^ ^]^ with a high scaling of $O(N^{8})$ as a price to pay. Clearly, this limits the applicability of CCSDT to small molecules.^[^ ^6^, ^7^, ^8^, ^34^, ^36^, ^37^ ^]^ Among the various cost‐effective approximations in the context of CCSD, the density‐fitting approaches such as use of Cholesky decomposition (CD),^[^ ^38^, ^39^, ^40^, ^41^, ^42^ ^]^ resolution‐of‐identity (RI)^[^ ^39^, ^41^, ^43^, ^44^ ^]^ techniques, have shown to gain significant speedup in CCSD with minimal reduction in accuracy as the decomposition of the electron repulsion integrals (ERI) greatly reduces the storage requirements which is the cost‐determining factor as the four‐index tensors (ERIs and $T_{2}$ tensors) are the biggest tensors in the CCSD computations. In contrast, the cost‐determining factor in CCSDT computations being the storage and iterative computation of the six‐index $T_{3}$ tensors, the ERI decomposition techniques would not be as effective in cost‐reduction as it turns out to be at CCSD level, although, such attempts have also been reported.^[^ ^45^ ^]^ Another way to improve performance is use of reduced precision (SP instead of DP), which satisfactorily maintains the accuracy with significantly lowered computational cost of CCSD and various EOM‐CCSD variants as was illustrated by Krylov and coworkers.^[^ ^46^, ^47^, ^48^ ^]^ The choice between DP and SP becomes crucial. A recent resurgence of interest in SP, driven by Graphic Processing Unit (GPU)^[^ ^49^, ^50^, ^51^, ^52^, ^53^, ^54^, ^55^, ^56^ ^]^ advantages, has led to research on mixed‐precision (MP) algorithms, particularly in integral calculations within Hartree–Fock and density functional theory. In MP, the equations are first converged within SP algorithm and then only limited additional steps are required for further convergence using DP. Investigations emphasize the inadequacy of pure SP for specific calculations, advocating for a DP or MP approach, especially in non‐iterative methods like second order Møller– Plesset (MP) perturbation theory (PT) (popularly known as MP2) and CCSD(T) (CCSD with perturbative triples correction), particularly, for gradients and properties. However, as far as energies are concerned, the errors of SP algorithm relative to DP are very insignificant (of the order of 1∼10 microhartrees) at CCSD level. In our recent implementation of SF‐EOM‐CCSDT method, we found that even at CCSDT level, the SP errors relative to DP for energies as well as energy‐gaps^[^ ^35^ ^]^ are just about 10 microhartrees (and sometimes, even smaller)! Yet another way of cost‐reduction is employing the FNO approach. NOs are defined as eigenvectors of a state one‐particle density matrix (DM). Pioneering investigations into NOs have extensively exploited them in various applications for constructing compact wavefunctions in configuration interaction (CI),^[^ ^57^, ^58^, ^59^, ^60^, ^61^, ^62^, ^63^ ^]^ multiconfigurational self‐consistent field (MCSCF),^[^ ^64^ ^]^ and CC^[^ ^65^, ^66^, ^67^ ^]^ methods. Löwdin illustrated that NOs result in faster convergence of the CI wavefunction expansion than HF MOs. Later, Barr and Davidson introduced FNOs,^[^ ^68^ ^]^ which keep the occupied orbitals at their HF values while transforming only the virtual orbitals. This ensures that both the reference and correlation energies remain unchanged throughout the transformation. Further Shavitt^[^ ^69^ ^]^ et al. demonstrated the advantages of the NOs over the HF orbitals through a thorough investigation of energy convergence in water. Several methods for generating NOs have been explored, including perturbation theory criteria and iterative variance, as well as using less computationally demanding method like MP2. The use of FNO in truncating PT and CC was pioneered by Bartlett and coworkers,^[^ ^65^, ^66^, ^67^ ^]^ demonstrating the efficacy of FNO‐based truncation in achieving accurate results with reduced computational cost. In *Q‐CHEM*,^[^ ^11^ ^]^ Krylov and coworkers implemented the FNO (computed at MP2 level) formulation within CCSD and EOM‐CCSD methods and also extended it to open‐shell (OS) references—the OSFNO technique for the spin‐flipping (SF) excited states within EOM‐CCSD formalism.^[^ ^70^, ^71^ ^]^ Both, the FNO technique and employing the SP algorithm sound promising candidates in cost‐reduction of conventional CCSDT (the one that uses the DP algorithm). In this article, we present some pilot applications of FNO‐based CCSDT method implemented within *Q‐CHEM* ^[^ ^11^ ^]^ with DP as well as with SP algorithms in the new coupled‐cluster suite (ccman2).^[^ ^72^ ^]^ In the next section, we present an outline of the CCSDT theory and briefly present the FNO approach and extrapolation of the same to give XFNO method. We present the computational details of the applications discussed in this article in Section 3, which is followed by the Section 4 in which we discuss the performance of FNO‐CCSDT relative to CCSDT using water molecule as test example and then present some pilot applications for illustrating accuracy of FNO‐ and XFNO‐CCSDT relative to CCSDT for total energies, energy‐gaps. We also present numerical estimates for force constants and harmonic vibrational frequencies of some diatomic molecules. Finally, we summarize our findings in the “Conclusions” section.

## 2 Theory

### 2.1 CCSDT Method

The essential working equations of the CCSDT method is well summarized below. In CC theory, the exact ground state wavefunction, $\Psi_{0}$ is obtained by action of exponential wave‐operator on the reference wavefunction, $\Phi_{0}$ as

(1) $$|\Psi_{0}\rangle =e^{\hat{T}}\left|\Phi_{0}\right\rangle$$

For closed‐shell molecules, $\Phi_{0}$ is the restricted Hartree–Fock determinant. (The method can be extended to lowest triplet /non‐singlet state of molecules by using a suitable restricted open‐shell or an unrestricted Hartree–Fock determinant as $\Phi_{0}$, as long as the corresponding HOMO and LUMO are well‐separated in energy). In CCSDT, the $\hat{T}$ operator is truncated so as to include up to three‐body excitations.

(2) $$\begin{array}{ccc} \hat{T} & = & {\hat{T}}_{1}+{\hat{T}}_{2}+{\hat{T}}_{3} \end{array}$$

(3) $$\begin{array}{ccc} {\hat{T}}_{1} & = & \sum_{i}^{occ}\;\;\sum_{a}^{virt}t_{i}^{a}a_{a}^{†}a_{i}\\ {\hat{T}}_{2} & = & \sum_{i<j}^{occ}\;\;\sum_{a<b}^{virt}t_{ij}^{ab}a_{a}^{†}a_{b}^{†}a_{i}a_{j}\\ {\hat{T}}_{3} & = & \sum_{i<j<k}^{occ}\;\;\;\sum_{a<b<c}^{virt}t_{ijk}^{abc}a_{a}^{†}a_{b}^{†}a_{c}^{†}a_{i}a_{j}a_{k} \end{array}$$

Consider the time independent Schrödinger wave Equation:

(4) $$\begin{array}{ccc} \hat{H}\left|\Psi_{0}\right\rangle & = & E_{0}\left|\Psi_{0}\right\rangle \end{array}$$

where, $E_{0}$ is the exact eigenvalue of the respective state. Now substituting Equations (1) in Equations (4), it follows

(5) $$\begin{array}{ccc} \hat{H}\left|\Psi_{0}\right\rangle & = & \hat{H}e^{\hat{T}}|\Phi_{0}\rangle =E_{0}e^{\hat{T}}\left|\Phi_{0}\right\rangle \end{array}$$

Premultiplying both the sides by $e^{-\hat{T}}$, and simplifying, we get

(6) $$\begin{array}{ccc} e^{-\hat{T}}\hat{H}e^{\hat{T}}\left|\Phi_{0}\right\rangle & = & {\left(\hat{H}e^{\hat{T}}\right)}_{C}|\Phi_{0}\rangle =\bar{H}|\Phi_{0}\rangle =E_{0}\left|\Phi_{0}\right\rangle \end{array}$$

(7) $$\begin{array}{ccc} \bar{H} & = & e^{-\hat{T}}\hat{H}e^{\hat{T}}\\ E_{CC} & = & E_{0}=\langle \Phi_{0}|\bar{H}\left|\Phi_{0}\right\rangle \end{array}$$

(8)

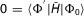

where $\bar{H}$ is the similarity transformed Hamiltonian. The 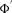 in the above Equation (8) are the excited determined obtained by replacing one or more occupied spin‐orbitals in $\Phi_{0}$ by virtual spin‐orbitals. By substituting 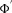 Equation (8) with $\Phi_{i}^{a}$, $\Phi_{ij}^{ab}$, and $\Phi_{ijk}^{abc}$, respectively, we obtain the equations for the amplitudes of ${\hat{T}}_{1}$, ${\hat{T}}_{2}$, and ${\hat{T}}_{3}$. ($\Phi_{i}^{a}$ is singly excited determinant obtained by replacing *i*th spin‐orbital by ath spin‐orbital, $\Phi_{ij}^{ab}$ is doubly excited determinant obtained by replacing ith and jth spin‐orbitals by ath and bth spin‐orbitals, and so on).

### 2.2 NOs and the FNO Approximation

Bartlett and coworkers^[^ ^65^, ^66^ ^]^ have well‐discussed the FNO‐CC method and have also explored the performance of CCSD(T) using FNO. We now briefly present the general formulation of FNO. The virtual orbitals are transformed into NOs by employing a series of simple operations using MP2 approximation to the DM which is defined as:

(9) $$\begin{array}{c} D_{ab}^{\left(2\right)}=1/2\sum_{cij}\frac{\left\langle cb||ij\rangle \langle ij||ca\right\rangle }{\in_{ij}^{cb}\in_{ij}^{ca}} \end{array}$$

where, indices i,j,k….. are for the occupied orbitals; and a,b,c….. are for the virtual orbitals, ⟨cb||ij⟩ are the anti‐symmetrized electron‐repulsion integrals in the molecular orbital basis and $\in_{ij}^{ab}$ is defined as

(10) $$\begin{array}{c} \in_{ij}^{ab}=f_{ii}+f_{jj}-f_{aa}-f_{bb} \end{array}$$

where, the $f_{pp}$ refers to the energy of p‐th canonical MOs (p‐th eigenvalue of the Fock operator). The transformation of HF virtual orbitals to NOs is done using the following series of steps.

(1) Starting with the canonical HF MOs, we compute $T_{2}$ amplitude at MP2 level.
(11) $$\begin{array}{c} t_{ij}^{ab}\left(MP2\right)=\frac{\left\langle ab||ij\right\rangle }{\in_{ij}^{ab}} \end{array}$$
(2) Then (within the virtual space), we generate the MP2 DM defined by Equation (9) and diagonalize it to generate the NOs. The (virtual) MO to NO coefficients are the the eigenvectors of the DM, whereas the occupation numbers are its eigenvalues.
(3) The NOs are then sorted in descending order of their occupation numbers.
(4) The NOs with high occupation numbers are retained (– treated active) for the CC calculations. The less important ones (that is, the last few ones—with low occupation numbers) are set aside and frozen.
(5) As an optional step, the active orbitals may be semi‐canonicalized, which can help faster convergence of CC iterations. The virtual blocks of one‐ and two‐electron integrals also need to be transformed to NO basis in this step. After this, the Fock matrix has block‐diagonal structure—frozen‐core block, active occupied block, and the active NO block. (The frozen NO block is not computed as it not required any further).

After computing the NOs, and freezing the desired fraction, we finally execute the CCSDT calculations in the truncated orbital space. There are two distinct truncation schemes of freezing the NOs within *Q‐CHEM*. The “percentage of total virtual orbitals” (POVO) scheme is self‐explanatory. For example, if POVO is set to 90%, it would freeze the highest 10% NOs and retain the remaining for the correlated calculations. The other scheme, “occupation threshold” (OCCT) uses the fraction of MP2 density to be retained while freezing the NOs. Both these schemes are elaborated by Krylov and coworkers.^[^ ^70^ ^]^

**Figure 1:**
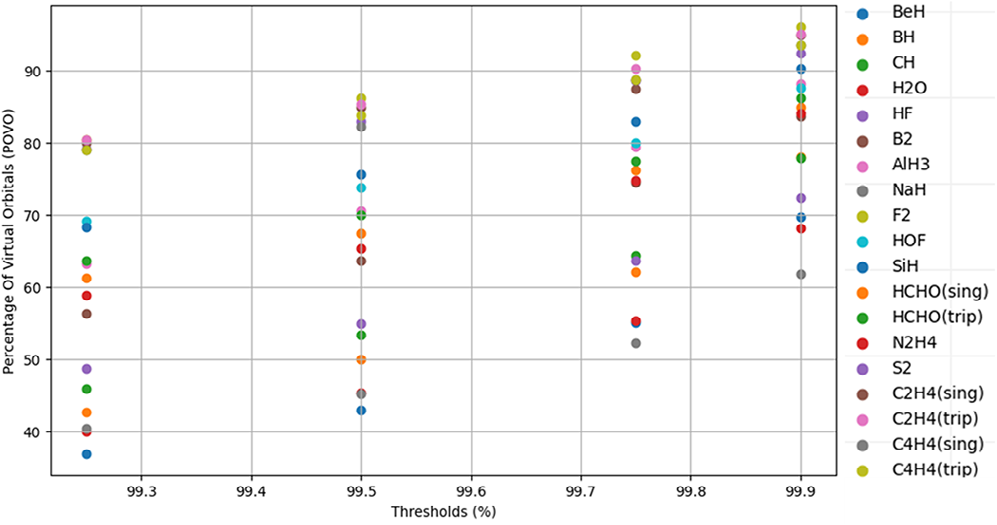
The POVO versus OCCT scatter plot.

Figure 1, presents the OCCT versus POVO plots for all the applications (molecules) discussed in this article. The POVO is defined as 100×AV/TV, where AV and TV are the numbers of active NOs and the total virtual orbitals, respectively. These are tabulated in Table 1. It is important to note that for a given OCCT value, there is a wide range of POVO (and vice versa). As a result, fixing POVO to some arbitrary value may not be *equally* good for all molecules as the fraction of the MP2 density retained at this value may significantly differ not only for different molecules, but also for different geometries of the same molecule. On the other hand, OCCT directly resembles with the fractional MP2 density retained after freezing the NOs and is more insightful about the quality of the computed results. Therefore, in this article, we use OCCT to define FNO thresholds. The OCCT=99.9% freezes less orbitals and is expected to give most accurate results. The speed‐ups for this threshold would be the lowest. Therefore, we discuss the accuracy and timings of FNO‐CCSDT OCCT=99.9% for all the molecules. The lower FNO thresholds would freeze more NOs, thereby reducing the accuracy. CCSDT being size‐extensive, the accuracy of FNO‐CCSDT increases almost linearly with FNO‐threshold. The linear extrapolation of energies computed for various FNO‐thresholds leads to the so‐called extrapolated (X)FNO method and can give more balanced and accurate results.

**Table 1:** Natural spin‐orbitals: Total versus active virtuals for various OCCT thresholds.

		No. of	No. of Active Natural Orbitals (AV)			
Molecule	Basis	Virtual Orbitals	OCCT	OCCT	OCCT	OCCT
	set	(all, TV)	99.9%	99.75%	99.50%	99.25%
BeH	cc‐pVQZ	165	115	91	71	61
BH	cc‐pVQZ	164	128	102	82	70
CH	cc‐pVQZ	163	127	105	87	75
$H_{2}O$	cc‐pVQZ	220	150	122	100	88
HF	cc‐pVTZ	78	68	62	56	52
$B_{2}$	cc‐pVTZ	110	92	82	70	62
$AlH_{3}$	cc‐pVTZ	136	120	108	96	86
NaH	cc‐pVTZ	84	52	44	38	34
$F_{2}$	cc‐pVTZ	102	98	94	88	82
HOF	cc‐pVTZ	130	114	104	96	90
SiH	cc‐pVTZ	81	73	67	61	55
HCHO(sing)	cc‐pVTZ	160	136	122	108	98
HCHO(trip)	cc‐pVTZ	160	138	124	112	102
$N_{2}H_{4}$	cc‐pVTZ	214	180	160	140	126
$S_{2}$	cc‐pVTZ	104	96	92	86	82
$C_{2}H_{4}$(sing)	cc‐pVDZ	80	76	70	68	64
$C_{2}H_{4}$(trip)	cc‐pVDZ	80	76	72	68	64
$C_{4}H_{4}$(sing)	cc‐pVDZ	124	116	110	102	98
$C_{4}H_{4}$(trip)	cc‐pVDZ	124	116	110	104	98

## 3 Computational Details

For the ease of discussion, we now introduce some conventions. We label the various methods as follows: $M_{1}$ refers to CCSDT, $M_{2}$ refers to FNO‐CCSDT, and OCCT = 99.9%. The results using FNO‐CCSDT with lower OCCT thresholds are used only to calculate the XFNO‐CCSDT results and are presented and explicitly mentioned only in the Supporting Information. The XFNO‐CCSDT method is explicitly mentioned (not abbreviated). The errors introduced by the use of SP instead of DP are rather insignificant (smaller than 10 microhartrees in $M_{1}$, $M_{2}$ as well as in XFNO‐CCSDT for all cases) and are not discussed separately. The use of SP versus DP is important only from the computational cost point of view and, therefore, is discussed in this article, in the context of computational timings only. In this context, the SP‐based results are explicitly labeled (e.g. $M_{1}\left(SP\right)$, $M_{2}\left(SP\right)$). Where the label (SP) is not used, it refers to the default DP algorithm. TE refers to total energy, whereas refers to the triplet–singlet gap, the gap between the lowest triplet state and the ground singlet state.

Development version of *Q‐CHEM* ^[^ ^11^ ^]^ package compiled using GNU compilers and openmp was used for all computations. The jobs for testing timings were run in sequential mode, whereas all the other jobs were run in multi‐threaded parallel mode. The basis sets used for various applications discussed in this article are as given in the Table 1. The equilibrium geometries of all the molecules the CCSDT and FNO‐CCSDT energy data at different OCCT thresholds required for XFNO extrapolation and for numerical estimation of the spectroscopic parameters are provided in the Supporting Information. The core orbitals were frozen during the post‐Hartree–Fock computations for all molecules, except in case of bond‐stretching trends in TEs and vertical TSGs of HF molecule.

## 4 Results and Discussion

### 4.1 Computational Timings

**Figure 2:**
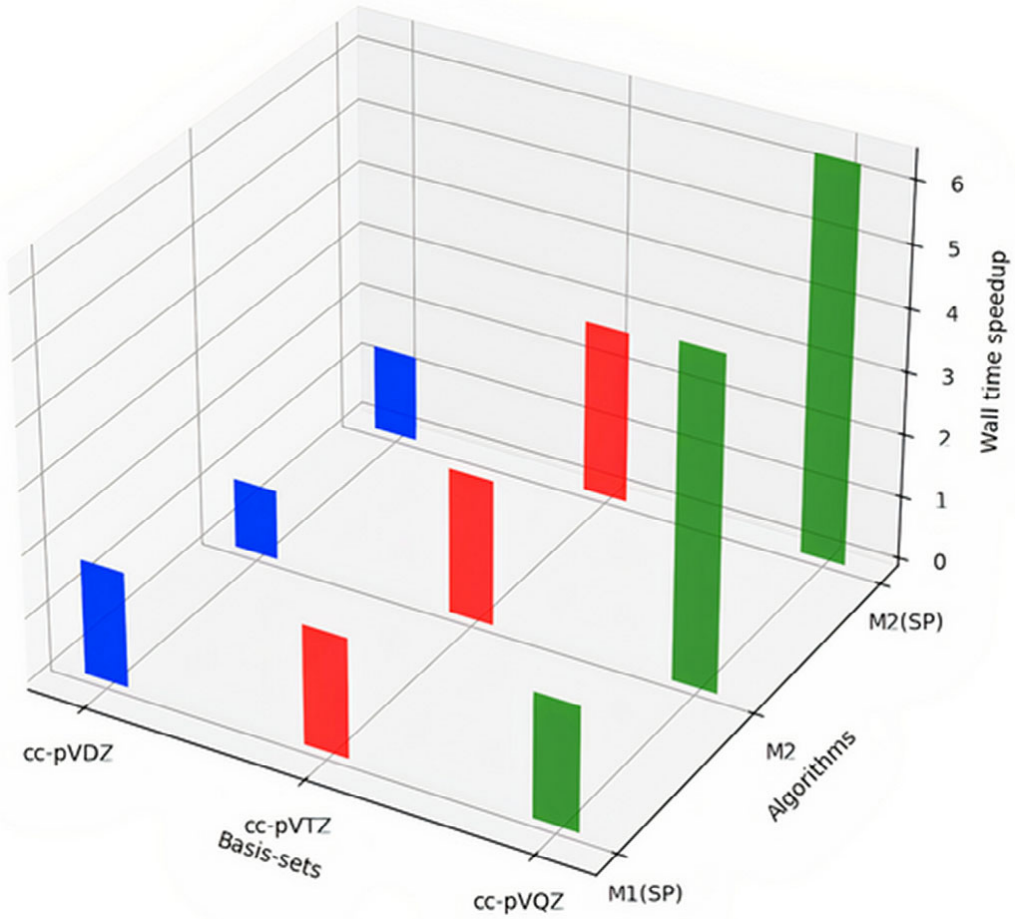
Speed‐ups in CCSDT(SP) and FNO‐CCSDT(DP and SP, OCCT=99.9%) relative to CCSDT(DP) for energy computation of water molecule using different basis sets.

The timings of $H_{2}O$ for the methods $M_{1}$ to $M_{2}\left(SP\right)$ and the basis sets, cc‐pVDZ, cc‐pVTZ, and cc‐pVQZ are presented in Table 2 and graphically in Figure 2. Relative to $M_{1}$, the $M_{1}\left(SP\right)$ shows a significant reduction in computational time approximately by 45% –49%, which is attributed to its reduced memory footprint by the factor of 2, regardless of the basis set employed. The speed‐up in FNO is due to reduction in the virtual orbital subspace, that accelerates the computation of costliest terms in the CCSDT iterations. Thus, the theoretical speed‐up in $M_{2}$ relative to $M_{1}$ would be approximately O(TV/AV)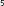. From 2, the theoretical speed‐ups in $M_{2}$ relative to $M_{1}$ (and, similarly in $M_{2}\left(SP\right)$ relative to $M_{1}\left(SP\right)$) come out to be 1.31, 2.54 and 6.35, respectively, for the cc‐pVDZ, cc‐pVTZ, and cc‐pVQZ bases. The actual speed‐up of X relative to Y is defined by the ratio of the timings: $t_{Y}/t_{X}$. For cc‐pVDZ, cc‐pVTZ, and cc‐pVQZ bases, $M_{2}$ is, respectively, 1.1, 2.31, and 5.28 times faster than $M_{1}$. For cc‐pVDZ, $M_{2}\left(SP\right)$ is rather slower (0.76 times faster) than $M_{1}\left(SP\right)$, due to very small computational size. However, for cc‐pVTZ and cc‐pVQZ, it is, respectively, 1.46 and 3.2 times faster relative to $M_{1}\left(SP\right)$. Although the actual speed‐ups in $M_{2}$ relative to $M_{1}$ are in good agreement with the theoretical ones, the performance of $M_{2}\left(SP\right)$ relative to $M_{1}\left(SP\right)$ does not seem to be encouraging at first sight. However, one needs to analyze the results and the theory more deeply to understand this behavior. As discussed in the theory, the FNO approach involves generation of NOs followed by transformation of integrals to the NO basis. These steps are computed using DP, irrespective of whether one employs DP or SP for the CCSDT iterations. Thus, these steps add to the computational timings significantly and turn out to be counter‐productive particularly for SP algorithm. However, upon comparing the $M_{2}\left(SP\right)$ timings with $M_{1}$ ones, we still observe significant speed‐up. For cc‐pVDZ, cc‐pVTZ, and cc‐pVQZ, $M_{2}\left(SP\right)$ is, respectively, 1.38, 2.76, and 6.34 times faster than $M_{1}$. These speed‐ups are nonetheless slightly larger than the speed‐ups of $M_{2}$ relative to $M_{1}$.

**Table 2:** Water molecule: Computational timings of various methods (in seconds).

Basis	Total Virtual(Active)	$M_{1}$	$M_{1}\left(SP\right)$	$M_{2}$	$M_{2}\left(SP\right)$
cc‐pVDZ	19(18)	426.87	234.543	384.560	309.326
cc‐pVTZ	53(44)	24247.57	12822.617	10496.78	8782.169
cc‐pVQZ	110(76)	8543265.7	4343297.3	1618042.8	1347518.3

### 4.2 FNO‐ and XFNO‐CCSDT Methods: Accuracy in Total Energies

Table 3 presents the $M_{1}$ TEs (in hartrees) and the relative errors in TEs (relative to $M_{1}$) computed using, $M_{2}$ and XFNO‐CCSDT for various molecules. The relative errors in FNO‐CCSDT decrease linearly with increasing OCCT from 99.25% to 99.9% and can be found in the Supporting Information. Using linear regression, these can be extrapolated to gives the XFNO‐CCSDT values.

**Table 3:** CCSDT total energies (in hartrees) and relative errors in $M_{2}$ and XFNO‐CCSDT energies (in millihartrees).

Molecules	Basis	$E_{\text{CCSDT}}$	$\delta E_{M_{2}}$	$\delta E_{\text{XFNO-CCSDT}}$
BeH	cc‐pVQZ	−15.19834	0.23677	0.16451
BH	cc‐pVQZ	−25.23533	0.50931	0.30456
CH	cc‐pVQZ	−38.41944	0.55394	0.23133
$H_{2}O$	cc‐pVQZ	−76.35664	0.98080	0.28329
B2	cc‐pVTZ	−49.29341	0.66869	0.12584
$AlH_{3}$	cc‐pVTZ	−243.76375	0.20928	0.00356
NaH	cc‐pVTZ	−162.42743	0.02099	0.01544
$F_{2}$	cc‐pVTZ	−199.29596	2.16889	1.37815
HOF	cc‐pVTZ	−175.33427	1.36551	1.18692
SiH	cc‐pVTZ	−289.54730	0.34845	0.08313
$N_{2}H_{4}$	cc‐pVTZ	−111.69511	1.30260	0.06176
$S_{2}$	cc‐pVTZ	−795.28790	1.28301	0.06724

Compared to $M_{1}$, both $M_{2}$ and XFNO‐CCSDT systematically overestimate total energies. The $M_{2}$ energies are higher than $M_{1}$ ones by 1.4 millihartrees or less for all molecules except $F_{2}$, for which the $M_{2}$ relative error is 2.16 millihartrees. XFNO significantly improves the accuracy. Except for $F_{2}$ and HOF, the XFNO‐CCSDT relative error are smaller than 1 millihartrees in all the molecules. For $F_{2}$ and HOF, the XFNO errors are, respectively, 1.4 and 1.2 millihartrees, which are smaller than the corresponding $M_{2}$ errors.

### 4.3 Ethene, Methanal, and Cyclobutadiene: Total Energies and Adiabatic Triplet–Singlet Gaps

Electronic structure and bonding‐patterns of ethene, methanal, and cyclobutadiene are interesting due to their potential importance in electronics, optics, or spintronics.^[^ ^73^, ^74^, ^75^ ^]^ The double bonds in these molecules play crucial role in their reactivity. All these molecules have non‐degenerate closed‐shell ground states, whereas their lowest triplet states are highly reactive. We present the TEs of the ground and the lowest triplet states, and the adiabatic TSGs of these molecules (excluding the zero‐point energy corrections) in Table 4. For ethene, $M_{2}$ overestimates ground state TE by 0.58 millihartrees, and the triplet state TE by 0.56 millihartrees, relative to $M_{1}$, resulting in marginal underestimation of the adiabatic TSG only by 0.021 millihartrees. On the other hand, XFNO‐CCSDT underestimates TEs as well as adiabatic TSGs relative to $M_{1}$. The absolute errors in ground and triplet state TEs are, respectively, 0.28 and 0.41 millihartrees resulting in the absolute error in the adiabatic TSG to be 0.12 millihartrees.

**Table 4:** Electronic energies and adiabatic TSGs (in Hartrees) of ethene, methanal and cyclobutadiene.

Molecule		$M_{1}$	$M_{2}$	XFNO‐CCSDT
$C_{2}$ $H_{4}$	$E_{S_{0}}\left(S_{0}=X^{1}A_{g}\right)$	−78.35633	−78.35575	−78.35662
	$E_{T_{1}}\left(T_{1}=1^{3}B_{1u}\right)$	−78.22442	−78.22385	−78.22482
	$\Delta E_{T_{1}-S_{0}}$	0.13192	0.13189	0.13179
HCHO	$E_{S_{0}}\left(S_{0}=X^{1}A_{1}\right)$	−114.33380	−114.33246	−114.33366
	$E_{T_{1}}\left(T_{1}=1^{3}A_{2}\right)$	−114.21470	−114.21377	−114.21482
	$\Delta E_{T_{1}-S_{0}}$	0.11909	0.11869	0.11884
$C_{4}$ $H_{4}$	$E_{S_{0}}\left(S_{0}=X^{1}A_{g}\right)$	−154.24168	−154.24031	−154.24225
	$E_{T_{1}}\left(T_{1}=1^{3}A_{2g}\right)$	−154.22199	−154.22058	−154.22249
	$\Delta E_{T_{1}-S_{0}}$	0.01969	0.01973	0.01976

For methanal, the TEs of $M_{2}$ are overestimated relative to $M_{1}$ by 1.34 and 0.94 millihartrees, respectively, for the ground and the triplet states, resulting in underestimation of the adiabatic TSG by 0.40 millihartrees. Whereas, for the ground state, XFNO‐CCSDT overestimates the TE by 0.134 millihartrees relative to $M_{1}$, it underestimates the triplet state TE by 0.11 millihartrees leading to underestimation of the adiabatic TSG by 0.25 millihartrees. In case of cyclobutadiene, the errors are slightly larger. $M_{2}$ overestimates the ground and triplet state TEs, respectively, by 1.37 and 1.41 millihartrees relative to $M_{1}$, leading to the overestimation of the adiabatic TSG by 0.05 millihartrees. The XFNO‐CCSDT on the other hand, underestimates the TEs, respectively, by 0.58 and 0.50 millihartrees relative to $M_{1}$, resulting in overestimation of the adiabatic TSG by 0.08 millihartrees.

### 4.4 Bond Stretching of Hydrogen Fluoride: Trends in Total Energies and Vertical Triplet–Singlet Gaps

**Figure 3:**
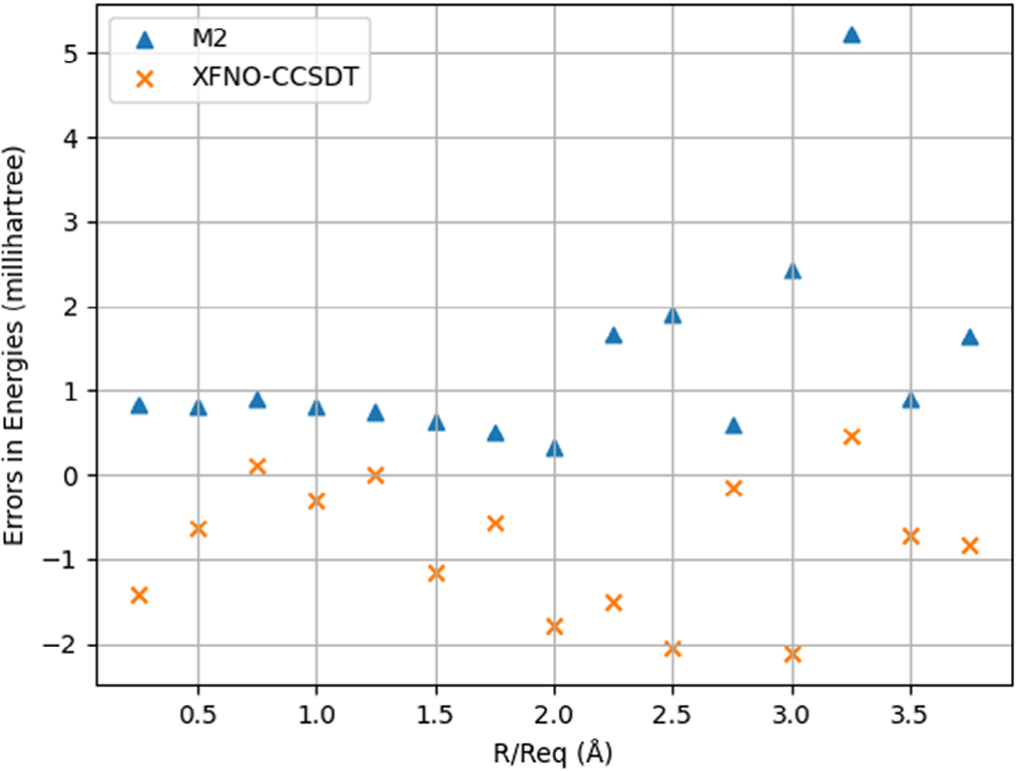
HF molecule: Bond‐stretching trends in TE errors relative to $M_{1}$.

**Figure 4:**
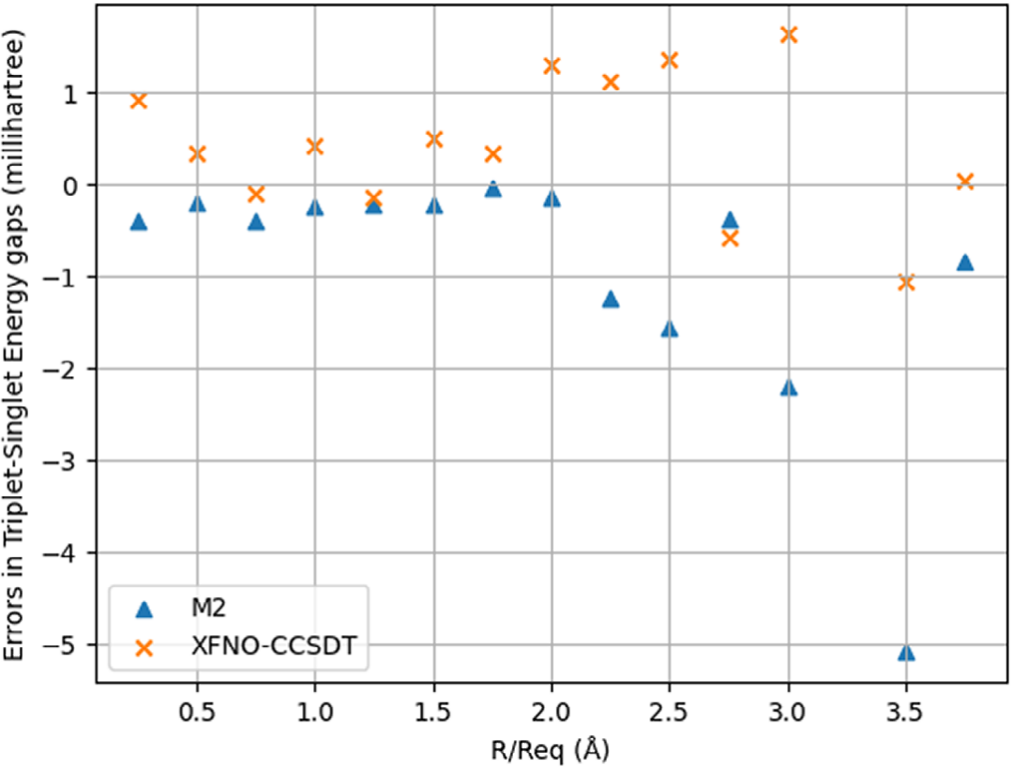
HF molecule: Bond‐stretching trends in TSG errors relative to $M_{1}$.

Figures 3 and 4 summarize the trends in the ground state TE and vertical TSG of HF molecule at different internuclear separations, in the scale of the ground state equilibrium bond‐length (0.915187Å). The numerical data can be found in the Supporting Information. Relative to $M_{1}$, the $M_{2}$ systematically overestimates the TEs, whereas systematically underestimates the vertical TSGs for all internuclear separations. XFNO‐CCSDT does not follow any such trend. The maximum absolute error (MAE) and non‐parallelity error (NPE) for $M_{2}$ TEs are, respectively, 5.21 and 4.87 millihartrees, which are reduced to, respectively, 2.12 and 2.58 millihartrees for XFNO‐CCSDT TEs. For vertical TSGs, the MAE, and NPE values for $M_{2}$ are 5.07 and 5.05 millihartrees, respectively, whereas for XFNO‐CCSDT, these are, respectively, 1.64 and 2.21 millihartrees. Inspite of lack of systematic trends in XFNO‐CCSDT errors (unlike in $M_{2}$), the NPEs in TE and vertical TSG are smaller than those of $M_{2}$, which indicates that the XFNO‐CCSDT relative errors are in general, smaller than the $M_{2}$ ones. The standard deviation in TEs is 1.23 millihartrees for $M_{2}$ and 0.81 millihartrees for XFNO‐CCSDT. For vertical TSGs, the standard deviation is 1.31 millihartrees for $M_{2}$ and 0.75 millihartrees for XFNO‐CCSDT.

### 4.5 Spectroscopic Parameters of Some Diatomic Molecules

We now test the accuracy various CCSDT variants in obtaining spectroscopic parameters, namely, the force constant (k) and fundamental vibrational frequencies ($\nu_{vib}$) of CO and HF molecules as test examples. Rigorous computation of these parameters would require computation of gradients and hessians, which is beyond the scope of the current work. However, computation of single‐point energies in the neighborhood of the minimum of the potential energy curve can enable one to estimate these parameters numerically. For our analysis, we start with the CCSD/cc‐pVDZ optimized equilibrium bond lengths of both molecules. We neglect the effect of change‐in‐electron‐correlation on the equilibrium bond lengths in our analysis. We use five‐point stencil formula^[^ ^76^ ^]^ to calculate numerical second‐order derivative:

(12) $$f^{\prime \prime }\left(x\right)\approx \frac{-f(x+2h)+16f(x+h)-30f(x)+16f(x-h)-f(x-2h)}{12h^{2}}$$

Using x as $R_{eq}$ and $h=0.05\;R_{eq}$ in the above formula, we compute the total energies (at CCSDT, FNO‐CCSDT, and XFNO‐CCSDT levels) at the given five internuclear separations to obtain the force constant as the second derivative in Equation (12). Using this force constant and the reduced mass of the given molecule, one can readily calculate the harmonic vibrational frequency. The spectroscopic parameters obtained this way, for the molecules, CO and HF are presented in Table 5.

**Table 5:** Numerical estimates for spectroscopic parameters of some diatomic molecules.

Molecule	Method	k ($Nm^{-1}$)	$\nu_{vib}$ ($cm^{-1}$)
	$M_{1}$	1839.19	2134.30
CO	$M_{2}$	1842.64	2136.30
	XFNO‐CCSDT	1842.60	2136.27
	Expt.^a^	1860	2143.3
	$M_{1}$	973.49	4156.43
HF	$M_{2}$	974.427	4158.09
	XFNO‐CCSDT	973.39	4156.19
	Expt.^b^	965	4138

^a)^ see Ref [77].

^b)^ see Ref [78, 79].

For *CO*, both $M_{2}$ and XFNO‐CCSDT overestimate the force constant relative to $M_{1}$ only by $3\;{\mathrm{Nm}}^{-1}$, resulting in overestimation of the vibrational frequency just by $2\;{\mathrm{cm}}^{-1}$. For HF, $M_{2}$ overestimates the force constant by just $1\;{Nm}^{-1}$ thereby overestimating the vibrational frequency by $1.5\;{\mathrm{cm}}^{-1}$. In this case, XFNO‐CCSDT error relative to $M_{1}$ is rather negligible for force constant, and hence, the vibrational frequency too.

## 5 Conclusions

We have presented our comprehensive study of FNO‐CCSDT and XFNO‐CCSDT methods for computing energies, energy gaps, and a few spectroscopic parameters of molecules and have discussed the accuracy and timings relative to the conventional CCSDT method. Relative to the conventional DP algorithm, SP reduces the computational cost of CCSDT iterations by almost 50%. However, when FNO and SP are used together, the speed‐up is majorly due to FNO, because the NOs are generated within the DP algorithm even if SP is employed during CCSDT. The use of SP does not affect the accuracy and should be preferred to DP wherever possible.

Although FNO‐CCSDT with a sufficiently larger threshold (OCCT = 99. 9%) can provide accurate results, additional computation of FNO‐CCSDT at several lower thresholds enables extrapolation to XFNO‐CCSDT values, which provide enhanced accuracy. The standard deviation calculated for the complete data discussed in this article (energies of all molecules, all geometries, all states) is 0.922 millihartrees for FNO‐CCSDT(with OCCT = 99.9%), which reduces to 0.614 millihartrees for XFNO‐CCSDT. Both these are within the CCSDT error range of $0.5∼\;1\;\mathrm{kcal}.\mathrm{mo}l^{-1}$. The numerical estimates on spectroscopic parameters of diatomic molecules provided by both these methods are also good with error of $∼\;2\;{\mathrm{Nm}}^{-1}$ for force constants and of $2\;{\mathrm{cm}}^{-1}$ for the vibrational frequency. In short, both FNO‐ and XFNO‐CCSDT approaches can be promising cost‐effective alternatives to the conventional CCSDT.

## Conflict of Interest

The authors declare no conflict of interest.

## Supporting information

Supporting Information

## Data Availability

The data that support the findings of this study are available in the supplementary material of this article.
