# Supplementary material for: Frozen Natural Orbitals‐Based Coupled‐Cluster Singles, Doubles, and (full) Triples ‐ A Computational Study
Source: Chem Asian J. 2025 Jun 6;20(14):e00472. doi: 10.1002/asia.202500472 (PMC12282650; doi:10.1002/asia.202500472)
Supplement: Supplementary file 1 — Supporting Information [file ASIA-20-e00472-s001.docx]

**Supplementary Information**

**for the manuscript**

**entitled**

**Frozen Natural Orbitals based Coupled-Cluster Singles, Doubles and (full) Triples - A Computational Study**

Manisha and Prashant Uday Manohar*

Department of Chemistry, BITS-PILANI, Pilani campus 333031

E-mail: [pumanohar@pilani.bits-pilani.ac.in](mailto:pumanohar@pilani.bits-pilani.ac.in)

All the distances are in angstroms, angles and dihedral angles are in degrees.

**1) Computational Timings**

Geometry of H2O for computation of timing jobs at cc-pV(DZ,TZ, and QZ) bases.

$molecule

0 1

O

H 1 1.0

H 1 1.0 2 100.

$end

Nuclear Repulsion Energy = 8.81223127 hartrees

SCF energy = -76.02128622 hartees

**2) The XFNO-CCSDT approach for total**

**energies**

Geometries of molecules computing at different occupation threshold (99.25%, 99.5%, 99.75%, and 99.9%)

$molecule

0 2

Be

H 1 1.357953

$end

Nuclear Repulsion Energy = 1.55874971 hartrees

SCF energy = -15.15334379 hartrees.

`````````````````````````````````````````````

$molecule

0 1

B

H 1 1.254792

$end

Nuclear Repulsion Energy = 2.10862522 hartrees.

SCF energy = -25.13091701 hartrees.

````````````````````````````````````````````````

$molecule

0 2

C

H 1 1.1198

$end

Nuclear Repulsion Energy = 2.83538423 hartrees

SCF energy = -38.28373482 hartrees

-------------------------------

$molecule

0 1

O

H 1 1.0

H 1 1.0 2 100.

$end

Nuclear Repulsion Energy = 8.81223127 hartrees

SCF energy = -76.05806775 hartees

-------------------------------

$molecule

0 1

H

F 1 0.915187

$end

Nuclear Repulsion Energy =5.20395821 hartrees

SCF energy = -100.05809154 hartrees

-----------------------------

$molecule

0 3

B

B 1 1.509914

$end

Nuclear Repulsion Energy = 8.76171111 hartrees.

SCF energy = -49.08254991 hartrees.

----------------------------

$molecule

0 1

Al 0.000000 0.000000 0.000000

H 0.000000 1.580500 0.000000

H 1.368753 -0.790250 0.000000

H -1.368753 -0.790250 0.000000

$end

Nuclear Repulsion Energy = 13.63775655 hartrees

SCF energy = -243.64430356 hartrees

---------------------

$molecule

0 1

Na

H 1 1.923706

$end

Nuclear Repulsion Energy = 3.02590381 hartrees

SCF energy = -162.39083783

------------

$molecule

0 1

F

F 1 1.432048

$end

Nuclear Repulsion Energy = 29.93150654 hartrees

SCF energy = -198.74912471

-------------

$molecule

0 1

O

H 1 0.972994

F 1 1.441630 2 97.454430

Nuclear Repulsion Energy = 33.36693892 hartrees

SCF energy = -174.80308981

$end

----------------

$molecule

0 2

Si

H 1 1.538658

$end

Nuclear Repulsion Energy = 4.81489776 hartrees

SCF energy = -289.43880808

-----------------

$molecule

0 1

C

H 1 1.117767

H 1 1.117767 2 115.299921

O 1 1.210367 2 122.350039 3 180.000000 0

$end

Nuclear Repulsion Energy = 31.09715806 hartrees

SCF energy = -113.91036558

------------------

$molecule

0 1

N

H 1 1.028125

H 1 1.028125 2 101.062769

N 1 1.482496 2 102.946993 3 106.212181 0

H 4 1.028125 1 102.946993 2 75.234036 0

H 4 1.028125 1 102.947002 2 -180.000000 0

$end

Nuclear Repulsion Energy = 40.87574639 hartrees

SCF energy = -111.21565004

--------------------

$molecule

0 3

S

S 1 1.927282

$end

Nuclear Repulsion Energy = 70.29037058 hartrees

SCF energy = -794.91780404

Table: S1 The energies of FNO-CCSDT at differnt OCCT threshold used for extrapolation of XFNO-CCSDT method.

| **Molecule** | **OCCT=99.25%** | **OCCT=99.50%** | **OCCT=99.75%** | OCCT=99.90% |
| --- | --- | --- | --- | --- |
| BeH | -15.19811 | -15.19795 | -15.19769 | -15.19753 |
| BH | -25.23482 | -25.23421 | -25.23375 | -25.23296 |
| CH | -38.41888 | -38.41808 | -38.41709 | -38.41616 |
| H_2_O | -76.35565 | -76.35404 | -76.35121 | -76.34804 |
| HF | -100.34452 | -100.34757 | -100.34941 | -100.35056 |
| B_2_ | -49.29274 | -49.29174 | -49.29026 | -49.28885 |
| AlH_3_ | -243.76354 | -243.76312 | -243.76262 | -243.76198 |
| NaH | -162.42741 | -162.42737 | -162.42729 | -162.42720 |
| F_2_ | -199.29379 | -199.29022 | -199.28297 | -199.27434 |
| HOF | -175.33291 | -175.33034 | -175.32511 | -175.31891 |
| SiH | -289.54695 | -289.54654 | -289.54595 | -289.54523 |
| N_2_H_4_ | -111.69381 | -111.69172 | -111.68838 | -111.68489 |
| S_2_ | -795.28662 | -795.28518 | -795.28200 | -795.27938 |

**3) Formaldehyde, Ethene, and Cyclobutadiene: Total Energies and Adiabatic Triplet-Singlet Gaps**

Formaldehyde optimized singlet geometry

$molecule

0 1

C

H 1 1.117767

H 1 1.117767 2 115.299921

O 1 1.210367 2 122.350039 3 180.000000 0

$end

Nuclear Repulsion Energy = 31.09715806 hartrees

SCF energy = -113.91036558

Formaldehyde optimized singlet geometry

$molecule

0 3

C

H 1 1.094027

H 1 1.094027 2 125.046510

O 1 1.331892 2 117.476745 3 -180.000000 0

$end

Nuclear Repulsion Energy = 29.22371588 hartrees

SCF energy = -113.83686518

Ethylene optimized triplet state geometry

$molecule

0 3

C

H 1 1.092161

H 1 1.092161 2 119.435357

C 1 1.547598 2 120.282321 3 180.000000 0

H 4 1.092161 1 120.282317 2 -180.000000 0

H 4 1.092161 1 120.282317 2 0.000000 0

$end

Nuclear Repulsion Energy = 30.74478681 hartrees

SCF energy = -77.9403073991 hartrees

Ethylene optimized singlet state geometry

$molecule

0 1

C

H 1 1.095776

H 1 1.095776 2 117.015116

C 1 1.350554 2 121.492442 3 -180.000000 0

H 4 1.095776 1 121.492442 2 -180.000000 0

H 4 1.095776 1 121.492442 2 0.000000 0

$end

Nuclear Repulsion Energy = 32.96609408 hartrees

SCF energy = -78.03865640 hartrees

Cyclobutadiene:

CCSD(T)/cc-pVTZ optimized

Singlet (X^1A_g) geometry; point group: D2h

Z matrix input:

C

C 1 ccs

C 2 ccd 1 ccc

C 3 ccs 2 ccc 1 dihh

H 1 hc 2 hccs 3 dihc

H 2 hc 3 hccd 4 dihc

H 3 hc 4 hccs 1 dihc

H 4 hc 1 hccd 2 dihc

ccs 1.566000

ccd 1.343000

hc 1.074000

ccc 90.000

hccs 134.910

hccd 135.090

dihh 0.000

dihc 180.000

Nuclear Repulsion Energy = 98.8821560064 hartrees

Cyclobutadiene:

CCSD(T)/cc-pVTZ optimized

Triplet (^3A_2g) geometry; point group: D4h

Z matrix input:

C

C 1 cc

C 2 cc 1 ccc

C 3 cc 2 ccc 1 dihc

H 1 hc 2 hcc 3 dihh

H 2 hc 3 hcc 4 dihh

H 3 hc 4 hcc 1 dihh

H 4 hc 1 hcc 2 dihh

cc 1.439000

ccc 90.000

ccc 90.000

dihc 0.000

hc 1.073000

hcc 135.000

dihh 180.000

Nuclear Repulsion Energy = 99.4931915115 hartrees

Table: S2 Total Energies of CCSDT(SP) and FNO-CCSDT(SP) methods.

| **Molecules** | **M_1_(SP)** | **M_2_(SP)** |
| --- | --- | --- |
| C_2_H_4_(singlet) | -78.35633 | -78.35575 |
| C_2_H_4_(triplet) | -78.22441 | -78.22385 |
| HCHO(singlet) | -114.33379 | -114.33246 |
| HCHO(triplet) | -114.21470 | -114.21377 |
| C_4_H_4_(singlet) | -154.24167 | -154.24030 |
| C_4_H_4_(triplet) | -154.22199 | -154.22058 |

**4)Bond Stretching of Hydrogen Fluoride: Trends in Total Energies and Vertical Triplet-Singlet Gaps**

R_eq_ = 0.915187

Nuclear Repulsion Energy =5.20395821 hartrees

SCF energy = -100.05809154 hartrees (at R_eq_, singlet ground state)

SCF energy = -99.73087581 hartrees (at R_eq_, triplet state)

Table: S3 The energies tabulated for the ground state singlet geometry of HF.

| **R** | **NRE** | **E_SCF_** | **E_CCSDT_** | **E_CCSDT (SP)_** |
| --- | --- | --- | --- | --- |
| 0.25 R_eq_ | 20.81583 | -93.06790 | -93.34963 | -93.34963 |
| 0.50 R_eq_ | 10.40792 | -99.07731 | -99.35347 | -99.35347 |
| 0.75 R_eq_ | 6.93861 | -99.95591 | -100.23930 | -100.23930 |
| 1.00 R_eq_ | 5.20396 | -100.05809 | -100.35137 | -100.35137 |
| 1.25 R_eq_ | 4.16317 | -100.01225 | -100.31582 | -100.31582 |
| 1.50 R_eq_ | 3.46931 | -99.94405 | -100.25906 | -100.25905 |
| 1.75 R_eq_ | 2.97369 | -99.88092 | -100.20997 | -100.20997 |
| 2.00 R_eq_ | 2.60198 | -99.82796 | -100.17471 | -100.17471 |
| 2.25 R_eq_ | 2.31287 | -99.78488 | -100.15285 | -100.15285 |
| 2.50 R_eq_ | 2.08158 | -99.75010 | -100.14104 | -100.14103 |
| 2.75 R_eq_ | 1.89235 | -99.62762 | -100.13536 | -100.13538 |
| 3.00 R_eq_ | 1.73465 | -99.69948 | -100.13287 | -100.13287 |
| 3.25 R_eq_ | 1.60122 | -99.68124 | -100.13186 | -100.13187 |
| 3.50 R_eq_ | 1.48685 | -99.59138 | -100.13148 | -100.13149 |
| 3.75 R_eq_ | 1.38772 | -99.58014 | -99.17150 | -99.17149 |

Table: S4 XFNO-CCSDT energies and FNO-CCSDT energies are tabulated at different OCCT threshold.

| **R** | OCCT=99.25  % | OCCT=99.50  % | OCCT=99.75  % | OCCT=99.90  % | XFNO-CCSDT |
| --- | --- | --- | --- | --- | --- |
| 0.25 R_eq_ | -93.33903 | -93.34580 | -93.34753 | -93.34879 | -93.35104 |
| 0.50 R_eq_ | -99.34567 | -99.34951 | -99.35157 | -99.35265 | -99.35409 |
| 0.75 R_eq_ | -100.23271 | -100.23454 | -100.23699 | -100.23840 | -100.23920 |
| 1.00 R_eq_ | -100.34452 | -100.34757 | -100.34941 | -100.35056 | -100.35167 |
| 1.25 R_eq_ | -100.30986 | -100.31133 | -100.31385 | -100.31507 | -100.31582 |
| 1.50 R_eq_ | -100.25057 | -100.25470 | -100.25760 | -100.25841 | -100.26021 |
| 1.75 R_eq_ | -100.20081 | -100.20385 | -100.20700 | -100.20947 | -100.21054 |
| 2.00 R_eq_ | -100.16142 | -100.16757 | -100.17145 | -100.17437 | -100.17650 |
| 2.25 R_eq_ | -100.13555 | -100.14139 | -100.14915 | -100.15119 | -100.15436 |
| 2.50 R_eq_ | -100.11653 | -100.12552 | -100.13484 | -100.13914 | -100.14310 |
| 2.75 R_eq_ | -100.11967 | -100.12599 | -100.12801 | -100.13477 | -100.13551 |
| 3.00 R_eq_ | -100.09368 | -100.10671 | -100.11994 | -100.13043 | -100.13500 |
| 3.25 R_eq_ | -100.08794 | -100.09973 | -100.11584 | -100.12664 | -100.13140 |
| 3.50 R_eq_ | -100.08488 | -100.10774 | -100.12886 | -100.13057 | -100.13220 |
| 3.75 R_eq_ | -99.14868 | -99.15700 | -99.16337 | -99.16985 | -99.17233 |

Table: S5 XFNO-CCSDT(SP) and FNO-CCSDT(SP) energies are tabulated at different OCCT threshold.

| **R** | OCCT=99.25  % | OCCT=99.50  % | OCCT=99.75  % | OCCT=99.90  % | XFNO-CCSDT |
| --- | --- | --- | --- | --- | --- |
| 0.25 R_eq_ | -93.33903 | -93.34580 | -93.34753 | -93.34879 | -93.35104 |
| 0.50 R_eq_ | -99.34567 | -99.34951 | -99.35157 | -99.35265 | -99.35409 |
| 0.75 R_eq_ | -100.23271 | -100.23454 | -100.23699 | -100.23840 | -100.23920 |
| 1.00 R_eq_ | -100.34452 | -100.34757 | -100.34941 | -100.35055 | -100.35167 |
| 1.25 R_eq_ | -100.30986 | -100.31133 | -100.31385 | -100.31507 | -100.31582 |
| 1.50 R_eq_ | -100.25057 | -100.25470 | -100.25760 | -100.25841 | -100.26021 |
| 1.75 R_eq_ | -100.20081 | -100.20386 | -100.20700 | -100.20947 | -100.21054 |
| 2.00 R_eq_ | -100.16142 | -100.16757 | -100.17145 | -100.17437 | -100.17650 |
| 2.25 R_eq_ | -100.13555 | -100.14138 | -100.14915 | -100.15119 | -100.15436 |
| 2.50 R_eq_ | -100.11653 | -100.12551 | -100.13483 | -100.13914 | -100.14310 |
| 2.75 R_eq_ | -100.11967 | -100.12771 | -100.13002 | -100.13480 | -100.13551 |
| 3.00 R_eq_ | -100.09368 | -100.10671 | -100.11994 | -100.13043 | -100.13500 |
| 3.25 R_eq_ | -100.08791 | -100.09966 | -100.11577 | -100.12666 | -100.13140 |
| 3.50 R_eq_ | -100.08488 | -100.10775 | -100.12889 | -100.13058 | -100.13220 |
| 3.75 R_eq_ | -99.14867 | -99.15702 | -99.16339 | -99.16985 | -99.17233 |

Table: S6 Triplet-Singlet Gaps for CCSDT, FNO-CCSDT at different OCCT threshold, and XFNO-CCSDT using double precision.

| **R** | **CCSDT** | OCCT=99.25% | OCCT=99.50% | OCCT=99.75% | OCCT=99.90% | XFNO-CCSDT |
| --- | --- | --- | --- | --- | --- | --- |
| 0.25 R_eq_ | 0.56597 | 0.56558 | 0.56509 | 0.56692 | 0.56111 | 0.56689 |
| 0.50 R_eq_ | 0.51243 | 0.51225 | 0.51203 | 0.51155 | 0.51005 | 0.51279 |
| 0.75 R_eq_ | 0.46264 | 0.46224 | 0.46215 | 0.46125 | 0.46091 | 0.46254 |
| 1.00 R_eq_ | 0.37961 | 0.37939 | 0.37964 | 0.37871 | 0.37757 | 0.38004 |
| 1.25 R_eq_ | 0.26896 | 0.26876 | 0.26822 | 0.26742 | 0.26735 | 0.26883 |
| 1.50 R_eq_ | 0.17101 | 0.17079 | 0.17058 | 0.17029 | 0.16809 | 0.17151 |
| 1.75 R_eq_ | 0.10010 | 0.10007 | 0.09845 | 0.09680 | 0.09554 | 0.10044 |
| 2.00 R_eq_ | 0.05351 | 0.05337 | 0.05104 | 0.04856 | 0.04401 | 0.05481 |
| 2.25 R_eq_ | 0.02583 | 0.02460 | 0.02315 | 0.01692 | 0.01298 | 0.02695 |
| 2.50 R_eq_ | 0.01114 | 0.00960 | 0.00540 | -0.00163 | -0.00892 | 0.01250 |
| 2.75 R_eq_ | 0.00408 | 0.00372 | -0.00283 | -0.00324 | -0.00761 | 0.00351 |
| 3.00 R_eq_ | 0.00095 | -0.00125 | -0.01016 | -0.02144 | -0.03235 | 0.00259 |
| 3.25 R_eq_ | -0.00033 | -0.00541 | -0.01465 | -0.02880 | -0.03848 | -0.00138 |
| 3.50 R_eq_ | -0.00080 | -0.00162 | -0.00177 | -0.02094 | -0.04168 | -0.00075 |
| 3.75 R_eq_ | -0.96078 | -0.96238 | -0.96730 | -0.97172 | -0.97791 | -0.96063 |

Table: S7 Triplet-Singlet Gaps for CCSDT, FNO-CCSDT at different OCCT threshold, and XFNO-CCSDT is presented using single precision.

| **R** | **CCSDT** | OCCT=99.25% | OCCT=99.50% | OCCT=99.75% | OCCT=99.90% | XFNO-CCSDT |
| --- | --- | --- | --- | --- | --- | --- |
| 0.25 R_eq_ | 0.56597 | 0.56558 | 0.56509 | 0.56692 | 0.56111 | 0.56689 |
| 0.50 R_eq_ | 0.51243 | 0.51225 | 0.51203 | 0.51155 | 0.51005 | 0.51279 |
| 0.75 R_eq_ | 0.46264 | 0.46224 | 0.46215 | 0.46125 | 0.46091 | 0.46254 |
| 1.00 R_eq_ | 0.37961 | 0.37939 | 0.37964 | 0.37871 | 0.37757 | 0.38004 |
| 1.25 R_eq_ | 0.26896 | 0.26876 | 0.26822 | 0.26742 | 0.26735 | 0.26883 |
| 1.50 R_eq_ | 0.17101 | 0.17079 | 0.17057 | 0.17029 | 0.16809 | 0.17151 |
| 1.75 R_eq_ | 0.10010 | 0.10007 | 0.09845 | 0.09680 | 0.09554 | 0.10044 |
| 2.00 R_eq_ | 0.05351 | 0.05337 | 0.05104 | 0.04856 | 0.04401 | 0.05481 |
| 2.25 R_eq_ | 0.02582 | 0.02460 | 0.02315 | 0.01692 | 0.01298 | 0.02695 |
| 2.50 R_eq_ | 0.01113 | 0.00959 | 0.00540 | -0.00163 | -0.00892 | 0.01250 |
| 2.75 R_eq_ | 0.00410 | 0.00374 | -0.00284 | -0.00324 | -0.00761 | 0.00351 |
| 3.00 R_eq_ | 0.00095 | -0.00125 | -0.01016 | -0.02144 | -0.03235 | 0.00259 |
| 3.25 R_eq_ | -0.00032 | -0.00548 | -0.01471 | -0.02888 | -0.03850 | -0.00138 |
| 3.50 R_eq_ | -0.00078 | -0.00165 | -0.00177 | -0.02094 | -0.04168 | -0.00075 |
| 3.75 R_eq_ | -0.96078 | -0.96236 | -0.96727 | -0.97172 | -0.97797 | -0.96063 |

**5)Spectrocopic parameters**

**i)** HF Molecule, R_eq_ = 0.918663

Nuclear Repulsion Energy = 5.18426768 hartrees (at R_eq_).

SCF energy = -100.01934594 hartrees (at R_eq_).

Table: S8 The total energies of M1, M2 and FNO-CCSDT at different threshold is tabulated below for employing five point stencil formula.

| **Methods** | **E(0.90 R_eq_)** | **E(0.95 R_eq_)** | **E(1.0 R_eq_)** | **E(1.05 R_eq_)** | **E(1.10 R_eq_)** |
| --- | --- | --- | --- | --- | --- |
| M1=CCSDT | -100.33999 | -100.34899 | -100.35137 | -100.34900 | -100.34326 |
| M2=FNO-CCSDT, OCCT (99.9%) | -100.33913 | -100.34815 | -100.35055 | -100.34820 | -100.34247 |
| FNO-CCSDT,  OCCT (99.75%) | -100.33788 | -100.34696 | -100.34941 | -100.34718 | -100.34120 |
| FNO-CCSDT,  OCCT (99.5%) | -100.33596 | -100.34507 | -100.34757 | -100.34532 | -100.33969 |
| FNO-CCSDT,  OCCT (99.25%) | -100.33315 | -100.34209 | -100.34453 | -100.34235 | -100.33683 |

**ii)** CO molecule, R_eq_=1.13838

Nuclear Repulsion Energy = 22.31285346 hartrees (at R_eq_).

SCF energy = -112.77890495 hartrees (at R_eq_).

Table: S9 The total energies of M1, M2 and FNO-CCSDT at different threshold is tabulated below for employing five point stencil formula.

| **Methods** | **E(0.90 R_eq_)** | **E(0.95 R_eq_)** | **E(1.0 R_eq_)** | **E(1.05 R_eq_)** | **E(1.10 R_eq_)** |
| --- | --- | --- | --- | --- | --- |
| M1=CCSDT | -113.12091 | -113.14853 | -113.15570 | -113.14904 | -113.13329 |
| M2=FNO-CCSDT, OCCT (99.9%) | -113.12000 | -113.14751 | -113.15454 | -113.14772 | -113.13179 |
| FNO-CCSDT,  OCCT (99.75%) | -113.11821 | -113.14562 | -113.15258 | -113.14571 | -113.12975 |
| FNO-CCSDT,  OCCT (99.5%) | -113.11502 | -113.14232 | -113.14966 | -113.14222 | -113.12632 |
| OCCT (99.25%) | -113.11235 | -113.13969 | -113.14585 | -113.13906 | -113.12196 |
